# Endoanal Ultrasound in Perianal Crohn’s Disease

**DOI:** 10.3390/jcm14196867

**Published:** 2025-09-28

**Authors:** Mario Pagano, Francesco Litta, Angelo Parello, Angelo Alessandro Marra, Paola Campennì, Carlo Ratto

**Affiliations:** 1 Proctology and Pelvic Floor Surgery Unit, Ospedale Isola Tiberina—Gemelli Isola, 00186 Rome, Italy; francesco.litta@fbf-isola.it (F.L.); angelo.parello@fbf-isola.it (A.P.); angeloalessandromarr@libero.it (A.A.M.); paola.campenni@fbf-isola.it (P.C.); carloratto@tiscali.it (C.R.); 2 Department of Medicine and Translational Surgery, Catholic University of the Sacred Heart, 00168 Rome, Italy

**Keywords:** perianal Crohn’s disease, endoanal ultrasound, magnetic resonance imaging, fistula, abscess, disease activity, PEACE index

## Abstract

**Background:** Perianal Crohn’s disease (pCD) is one of the most disabling complications of inflammatory bowel disease, characterized by fistulas and abscesses that demand precise imaging for diagnosis, treatment planning, and follow-up. Magnetic resonance imaging (MRI) is considered the reference standard, but endoanal ultrasound (EAUS) with high-frequency 360° probes provide a readily available, cost-effective, and repeatable alternative. **Methods**: We performed a narrative review of the literature, evaluating studies on the EAUS technique, diagnostic applications, distinguishing features of Crohn’s-related fistulas, and comparative analyses with MRI. Consensus documents and structured reporting initiatives were also included. **Results**: EAUS provides high-resolution visualization of the anal sphincter complex and intersphincteric space, enabling the reliable detection of fistulas and abscesses. Characteristic features such as tract width > 4 mm, bifurcation, hyperechoic debris, the Crohn’s Ultrasound Fistula Sign (CUFS), and the rosary sign assist in differentiating Crohn’s from cryptoglandular fistulas. EAUS is well-suited for serial monitoring, perioperative seton guidance, and therapeutic decision-making. Emerging tools such as Doppler and shear wave elastography provide additional information on activity and fibrosis. MRI remains indispensable for supralevator disease, deep pelvic sepsis, and standardized activity indices. Comparative studies indicate similar sensitivity for simple fistulas, with MRI superior in complex cases; combining both modalities maximizes accuracy. **Conclusions**: EAUS is a practical and repeatable imaging tool that complements MRI in the multidisciplinary management of perianal Crohn’s disease. Advances such as 3D imaging, contrast enhancement, and elastography may enable validated activity scoring—for example, a future PEACE (Perianal Endosonographic Activity in Chron’s Evaluation) Index—further strengthening its role in longitudinal care.

## 1. Introduction

Crohn’s disease (CD) is a chronic, recurrent inflammatory bowel disease characterized by transmural inflammation and a heterogeneous clinical course, potentially affecting any part of the gastrointestinal tract. Among its extraintestinal and local complications, perianal Crohn’s disease (pCD) is one of the most disabling manifestations. Perianal involvement occurs in approximately 20–30% of patients, although the reported prevalence varies between 14% and 54%, depending on the population, disease location, and follow-up duration [1]. In up to one-fifth of cases, perianal fistulas or abscesses may precede the diagnosis of intestinal disease, and in rare situations may represent the first and only manifestation for years [2].

Clinical presentations of pCD also include perianal fissures, mucosal ulcers, abscesses, fistulas, strictures, skin tags, and, in advanced cases, rectovaginal or rectourethral fistulas. These lesions often progress insidiously, and up to two-thirds of fistulas are relatively painless at onset [3]. The presence of perianal disease is strongly associated with a more aggressive phenotype and earlier need for surgery [4]. The cumulative incidence of fistulizing disease increases over time: perianal fistulas are reported in 18% of patients by 10 years after diagnosis, rising to nearly one-quarter after three to four decades [5].

Beyond its clinical manifestations, perianal Crohn’s disease carries a substantial socio-economic burden. Patients with pCD experience higher hospitalization rates, repeated surgical procedures, and increased use of advanced therapies, translating into considerable direct health-care costs and loss of productivity [6]. Furthermore, international data indicate geographical variability, with higher prevalence in Western countries but a rising incidence in Asian populations, likely reflecting environmental and genetic interactions [7].

The pathophysiology of CD involves transmural inflammation that predisposes to penetrating complications [8]. Perianal tracts in CD often differ from cryptoglandular fistulas: they may be multiple, branching, or bifurcated, frequently defy classical rules such as Goodsall’s rule, and are commonly associated with secondary extensions and abscesses [9]. Differentiating Crohn-related fistulas from cryptoglandular ones is clinically crucial, as therapeutic strategies diverge significantly.

The therapeutic landscape has evolved substantially with the introduction of biologics, especially anti-TNF (Tumor Necrosis Factor) agents [10], in combination with immunomodulators, antibiotics, other biologics, and surgical interventions, which have improved outcomes [11]. Nevertheless, pCD remains a significant determinant of disease burden, requiring accurate imaging to stratify severity, guide medical-surgical decisions, and monitor response.

In this complex clinical scenario, imaging has become an essential component of the multidisciplinary management of pCD. High-resolution modalities not only enable accurate classification of fistulas but also serve as objective tools to monitor treatment response and guide surgical timing. Both radiologists and colorectal surgeons increasingly rely on imaging findings to tailor medical therapy, optimize seton management, and decide when to attempt definitive closure procedures. Current guidelines recommend pelvic MRI as the reference standard for mapping fistulas and guiding surgery, supported by validated activity scores [12]. Endoanal ultrasound (EAUS) offers a complementary, cost-effective, and repeatable bedside technique for delineating tracts and detecting collections, particularly when MRI is unavailable or contraindicated. Despite significant progress, important gaps remain in the standardization of EAUS. Variability in technique, operator experience, and reporting practices currently limit its widespread adoption. The development of structured templates, integration of novel tools such as Doppler and shear wave elastography, and validation of EAUS-based activity indices represent key research priorities for the coming years.

In parallel with these advances, the management of inflammatory bowel disease has increasingly adopted a treat-to-target (T2T) strategy, in which therapeutic decisions are guided by objective markers of inflammation rather than symptoms alone [13]. Landmark trials such as CALM and STARDUST [14,15] demonstrated that tight control based on endoscopy and biomarkers can improve endoscopic outcomes compared with symptom-driven care, even if primary endpoints were not always met. This paradigm reflects the broader shift toward value-based healthcare, emphasizing early intervention, prevention of complications, and cost-effectiveness. Within this framework, EAUS emerges not merely as an alternative to MRI, but as a practical enabler of serial monitoring and personalized treatment strategies, complementing MRI in a T2T and multidisciplinary approach to perianal Crohn’s disease.

In this context, understanding the strengths and limitations of endoanal ultrasound in comparison to MRI is essential for optimizing care.

## 2. EAUS Technique

Endoanal ultrasound (EAUS), introduced by Law and Bartram in 1989 [16], is performed using high-frequency radial probes, typically in the 6–16 MHz range, equipped with 360° mechanical rotation. Modern systems enable the automated extraction of datasets, generating two-dimensional and three-dimensional data with field depths of up to 10 cm.

Before the procedure, careful inspection of the perianal region and a digital rectal examination (DRE) should be performed to assess anal sphincter tone and palpate lesions. The examination is generally well tolerated. Patients are positioned in the left lateral decubitus position, and no anesthesia or specific bowel preparation is usually required. The probe covered with a protective sheath and lubricated is introduced gently into the anal canal.

Standardized orientation is crucial for reproducibility [17]. By convention, the anterior wall is displayed at 12 o’clock, the left at 3 o’clock, the posterior at 6 o’clock, and the right at 9 o’clock. Sequential scans are obtained as the transducer is withdrawn from the rectal ampulla to the distal anal canal, enabling systematic visualization of three key planes:

- Deep plane: The proximal anal canal, characterized by the U-shaped sling of the puborectalis muscle.
- Intermediate plane: The hypoechoic internal anal sphincter, the perineal body, and the transverse perineal muscle.
- Superficial plane: The distal canal, including the submucosal portion of the external anal sphincter.

EAUS reliably identifies the main anatomical components of the sphincter complex and provides high-resolution images of fistulous disease. Fistula tracts appear as hypoechoic, tubular structures; the internal opening is usually recognized as a focal defect in the internal anal sphincter or a subepithelial breach connecting with a tract in the intersphincteric plane. Secondary extensions are classified by their anatomic location-whether intersphincteric, ischiorectal, or supralevator-and horseshoe tracts are defined by bilateral spread around the internal opening [18].

Variability in reporting has historically limited the reproducibility of EAUS. Recent international consensus has therefore emphasized the need for structured reporting: the 2024 SMART (Structured MRI and EAUS Anal Fistula Reporting Template) project, developed through a multidisciplinary Delphi process, defined a minimal dataset for standardized reports [19]. Key elements include description of the primary tract, internal opening(s), secondary extensions, collections, coexisting lesions, and sphincter morphology, ideally complemented by diagrammatic representation. Such templates enhance interdisciplinary communication, improve reproducibility, and support surgical planning.

In addition to EAUS, some centers have explored transperineal ultrasound (TPUS) as a noninvasive alternative. TPUS can be useful in patients with anal stenosis, severe pain preventing probe insertion, or when a less invasive, outpatient-friendly approach is desirable. While TPUS provides lower spatial resolution of the anal sphincter complex compared with EAUS, it can still identify fistulous tracts and collections and may serve as a complementary option in selected cases [20].

EAUS is generally safe, well-tolerated, and free of radiation or contrast-related risks. Contraindications are limited, mainly to severe anal stenosis or acute pain preventing probe insertion. As with other ultrasound techniques, interpretation is operator dependent, requiring adequate training and experience to achieve high diagnostic accuracy.

## 3. Diagnosis with EAUS

In the diagnostic work-up of perianal Crohn’s disease (pCD), EAUS is a pivotal tool for identifying and characterizing fistulas and abscesses [21,22,23,24]. Precise anatomical delineation and assessment of their relationship with the anal sphincter complex are essential for treatment planning and minimizing recurrence. EAUS provides high-resolution circumferential imaging of the anal canal, enabling the classification of fistulas as simple or complex and the identification of features that guide both surgical and medical management.

On EAUS, fistulous tracts typically appear as continuous hypoechoic linear structures in the subepithelial or intersphincteric space, often associated with a defect in the internal anal sphincter and considered a marker of active inflammation [25]. Small hyperechoic foci, corresponding to intraluminal gas, may also be observed. Internal openings are usually identified as focal hypoechoic disruptions abutting the internal sphincter.

**Figure 1:**
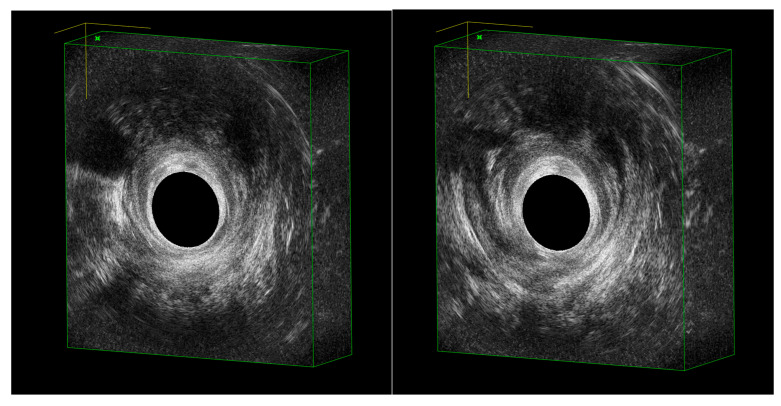
Hydrogen peroxide–enhanced EAUS of a Crohn’s transsphincteric fistula with internal opening at 6 o’clock, extending to the perineum and toward the right posterolateral vaginal wall, with tract delineation clarified by microbubbles.

To enhance visualization, contrast agents such as diluted hydrogen peroxide may be instilled through the external fistula opening [26]. Then, it generates hyperechoic microbubbles, which clarify the relationship of the fistula to the anal canal and sphincters. Prospective studies have shown that hydrogen peroxide-enhanced EAUS identifies the internal opening in over 90% of cases and demonstrates high concordance with surgical findings [27]. An illustrative example of hydrogen peroxide–enhanced EAUS of a Crohn’s transsphincteric fistula is presented in Figure 1.

Beyond fistulas, EAUS is valuable for detecting abscesses, which may be superficial in the perianal region or extend deeply as perirectal collections. Recognition is crucial since abscesses are strongly linked to fistula formation. Surgical drainage of an abscess without addressing an underlying fistula often leads to recurrence, underscoring the importance of accurate imaging [28]. While EAUS excels at identifying collections within its focal range, its performance is limited for very lateral or supralevator tracts.

The advent of three-dimensional EAUS has further improved the accuracy of tract mapping and internal opening detection, while maintaining patient tolerability and procedural simplicity [29]. When performed systematically and documented using structured reporting templates, EAUS yields reproducible, clinically actionable findings that integrate seamlessly into multidisciplinary care pathways (Table 1).

### Typical EAUS Signs of pCD

Differentiating Crohn’s-related fistulas from cryptoglandular fistulas is a central challenge, as management strategies diverge substantially. Over the past decade, several characteristic EAUS features have been described to aid this distinction. These signs are useful both when a perianal fistula represents the first manifestation of Crohn’s disease and when new fistulas arise in patients with established CD that may in fact be cryptoglandular.

One of the earliest systematic efforts was reported by Blom et al. [30], who studied 45 patients with Crohn’s disease and anal fistulas. They identified three features suggestive of Crohn’s etiology: bifurcation or secondary tracts, tract width greater than 3 mm, and the presence of hyperechoic debris. The authors proposed that the presence of at least two of these signs strongly indicated Crohn’s-related disease, although prevalence data were limited.

**Figure 2:**
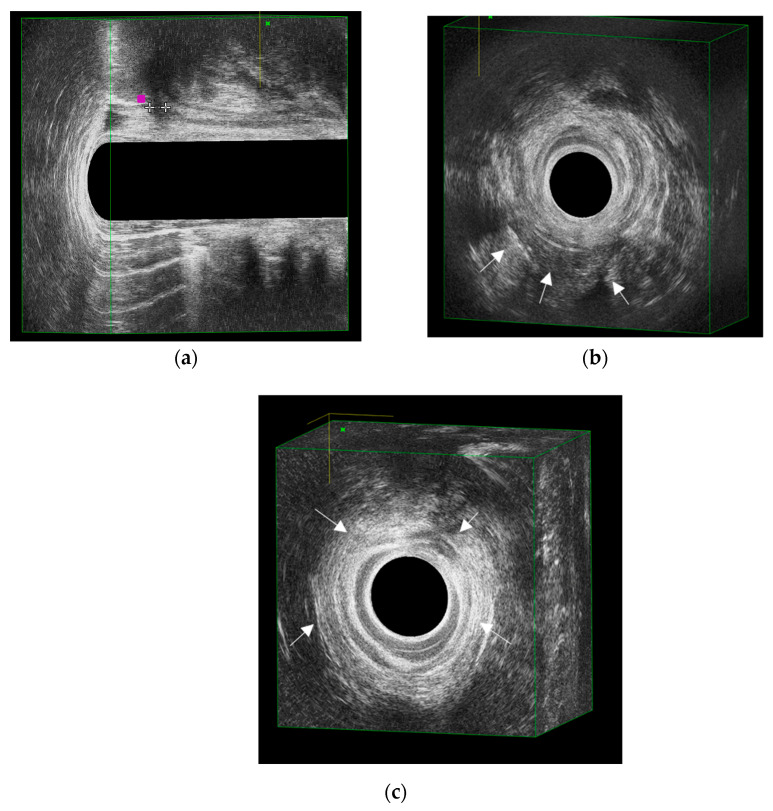
Representative EAUS features of Crohn’s-related perianal fistulas. (**a**) Tract width > 4 mm, visualized as a hypoechoic tubular structure exceeding the normal diameter of cryptoglandular tracts; (**b**) Crohn’s Ultrasound Fistula Sign (CUFS), defined as a hypoechoic tract surrounded by a thin hypoechoic rim and an outer hyperechoic halo; (**c**) Rosary sign, characterized by a discontinuous “rosary bead”-shaped hypoechoic halo in the intersphincteric space of the upper anal canal.

A more specific sonographic marker was later described by Zawadzki et al. [31] as the Crohn’s Ultrasound Fistula Sign (CUFS). CUFS is defined as a hypoechoic fistula tract with a thin hypoechoic rim, surrounded by a hyperechoic region (Figure 2b). In their series of 157 patients (29 with Crohn’s and 128 with cryptoglandular disease), CUFS demonstrated high specificity (98%) but only moderate sensitivity (69%) for Crohn’s-related fistulas. Interobserver agreement was good (κ = 0.77), and subsequent validation by Zbar et al. [32] confirmed similar specificity (97%), though with lower sensitivity (43%), reinforcing CUFS as a highly specific but not universal marker.

The contribution of tract width was further highlighted by Luglio et al. [33], who evaluated 158 patients, including 33 with Crohn’s disease. They analyzed four features—CUFS, bifurcation, debris, and tract width—and concluded that a diameter greater than 4 mm (Figure 2a) was the most reliable individual predictor of Crohn’s-related fistulas (sensitivity 81%, specificity 97%). Significantly, combining two or more features, such as CUFS and a tract of 4 mm, increased diagnostic specificity to 100%. Interobserver agreement was excellent (κ = 0.84), suggesting reproducibility in expert hands.

Another distinctive finding, the rosary sign, was described by de la Portilla et al. [34] as a discontinuous “rosary bead”-shaped hypoechoic halo encircling the intersphincteric space in the upper anal canal (Figure 2c). This feature was more frequent in Crohn’s fistulas and carried predictive value: patients with the rosary sign were more than twice as likely to have Crohn’s disease compared with those without it. While specificity was moderate (71%) and sensitivity was low (49%), its negative predictive value was high, suggesting that the absence of the sign argues against a diagnosis of Crohn’s. Detection rates were acceptable even among less experienced observers.

Overall, no single ultrasound sign is sufficient for reliable discrimination. Instead, combining features—such as tract width greater than 4 mm, bifurcation, debris, CUFS, and the rosary sign—provides the best diagnostic accuracy. In clinical practice, recognition of these features facilitates early identification of Crohn’s-related perianal disease, prevents unnecessary surgery, and prompts timely initiation of advanced medical therapy [33]. An overview of the pertinent literature addressing the characteristic EAUS findings in pCD is provided in Table 2.

## 4. Disease Activity and Management with EAUS

Once a diagnosis of fistulizing perianal Crohn’s disease (pCD) is established, the role of imaging extends beyond anatomy to monitoring activity, guiding treatment, and detecting remission or recurrence. Imaging findings, interpreted alongside clinical parameters, inform the choice between medical, surgical, or combined strategies, determine treatment endpoints, and support decisions on when to escalate or de-escalate therapy [35].

EAUS is particularly well-suited for serial monitoring because it is inexpensive, well-tolerated, and repeatable at the bedside. Changes in fistula caliber, internal echoes, or vascularity can be tracked across examinations. Such longitudinal assessments are valuable for timing drainage procedures, adjusting seton management [36], and optimizing biologic therapy [37]. In acute settings, EAUS also detects abscesses and secondary collections, ensuring that sepsis is drained before intensifying immunosuppressive therapy [38].

Several prospective and retrospective studies have highlighted the clinical utility of EAUS-guided decision-making [36,37,39]. In randomized trials, tailoring surgical or medical interventions to EAUS findings resulted in earlier biologic escalation, fewer re-interventions, and higher rates of fistula closure compared with standard care. For example, EUS-guided seton strategies improved outcomes by identifying persistent peri-seton inflammation, avoiding premature removal, and confirming quiescence before advancement flap or medical closure [40].

Technological refinements have expanded the scope of EAUS in activity assessment. Since fistula fibrosis is considered a marker of radiological healing [41], Hong N. et al. [42] investigated EAUS combined with shear wave elastography (SWE) for evaluating perianal fistula activity. Doppler assessment of vascularity and SWE parameters have been correlated with MRI-based indices, such as MAGNIFI-CD [43], and clinical scores, including the Perianal Disease Activity Index (PDAI) [44]. Increased vascular signals and reduced shear wave velocities have been associated with active inflammation, while higher elastic modulus values suggest fibrosis and healing. These objective markers complement grayscale findings, where pus and fibrosis may appear similarly hypoechoic. The Hong et al. study indicates that SWE combined with EAUS could provide an objective biomarker of activity, paralleling MRI-based scoring systems.

Prognostic application of EAUS is an emerging field and has been highlighted as a priority by international consensus groups [45]. Features of fistula complexity—such as high trans- or suprasphincteric extension, multiple openings, or associated abscesses—are already recognized predictors of poor healing [46]. Serial EAUS may further refine prognostication by identifying patients with negligible residual activity who can safely discontinue biologics, as shown in small retrospective cohorts [47]. Conversely, persistent hypoechoic tracts or Doppler signal despite clinical closure suggest ongoing activity and a high risk of recurrence if treatment is withdrawn [48].

Overall, EAUS provides practical advantages for integrating imaging into the day-to-day management of pCD. It enables rapid, inexpensive, and repeatable monitoring of activity, supports seton management, and complements MRI during treatment intervals. Looking ahead, two avenues of research may substantially enhance the accuracy and reproducibility of EAUS. First, the integration of artificial intelligence (AI) and machine learning could support less experienced operators by standardizing the identification of internal openings, classifying fistula types, and detecting subtle indicators of activity [49]. Early work in other imaging domains suggests that algorithm-assisted interpretation can reduce variability, improves physician confidence and reduces interpretation time [50,51]. Second, advanced EAUS modalities such as Doppler and shear wave elastography (SWE) require standardized acquisition protocols and validated cutoff values to enable reproducibility across centers and platforms. Recent studies have shown that SWE correlates with MRI-based indices such as MAGNIFI-CD and with clinical activity scores [42], underscoring its potential as an objective biomarker. With structured reporting and emerging adjuncts such as SWE and AI-assisted interpretation, EAUS is evolving from a purely anatomical into a combined anatomical and functional imaging tool for the longitudinal management of patients, thereby strengthening its role within treat-to-target strategies for perianal Crohn’s disease.

## 5. Comparative Roles: EAUS vs. MRI

Magnetic resonance imaging (MRI) is widely regarded as the reference standard for perianal Crohn’s disease, owing to its multiplanar capabilities, excellent soft tissue contrast, and ability to assess the entire pelvic floor, including supralevator and extrapelvic extensions [12,52]. MRI is particularly valuable for preoperative mapping, long-term monitoring, and as the basis for validated activity scores such as the Van Assche index and MAGNIFI-CD [53]. Limitations include high costs, reduced accessibility, and contraindications in patients with implanted devices or severe claustrophobia. In urgent settings, scheduling delays may hinder timely decision-making.

By contrast, endoanal ultrasound (EAUS) offers a readily available, low-cost, and radiation-free alternative that can be performed at the bedside, intraoperatively for seton management, or in outpatient clinics. Modern three-dimensional probes offer high-resolution imaging of the anal sphincter complex and intersphincteric space, allowing for the precise identification of internal openings, delineation of primary and secondary tracts, and detection of associated abscesses. EAUS is particularly useful for classifying fistulas in relation to the sphincters and for guiding intraoperative seton placement [54]. When enhanced with hydrogen peroxide, its accuracy in localizing internal openings approaches that of MRI [26]. Moreover, EAUS is the modality of choice for assessing sphincter integrity, especially in patients with prior anorectal surgery, as it provides essential information for surgical planning and evaluation of continence risk [55].

However, EAUS has intrinsic limitations: very lateral or supralevator tracts and complex secondary extensions are often beyond the field of view, and anal stenosis or severe pain may preclude probe insertion. Buchanan et al. reported that MRI-guided surgical planning was associated with a 75% reduction in postoperative recurrence compared with surgery without MRI guidance [56].

Comparative studies report variable results. Schwartz et al. Schwartz et al. found EAUS and examination under anesthesia (EUA) to be more accurate than MRI in some cases, with >90% concordance to surgical findings [23].

Orsoni et al. similarly showed EAUS to be the most sensitive modality among 22 patients with perianal Crohn’s disease [22]. In contrast, a meta-analysis by Siddiqui et al. found comparable sensitivities for EAUS and MRI (both 87%) but higher specificity for MRI (69% vs. 43%) [24]. More recent studies suggest that EAUS and MRI perform similarly for simple fistulas, whereas MRI retains superiority in complex and recurrent cases [57]. These variable results should be interpreted considering important methodological differences across studies. Heterogeneity in study populations—particularly the mix of simple versus complex fistulas—as well as variability in operator expertise and the reliance on earlier-generation ultrasound equipment likely account for discrepancies in reported sensitivity and specificity. For instance, the meta-analysis by Siddiqui et al. highlighted lower specificity for EAUS compared with MRI, but most included studies were performed before the widespread adoption of 3D technology or peroxide enhancement [24]. More recent data indicate that modern, high-frequency, 3D EAUS in expert hands achieves substantially higher accuracy, narrowing the gap with MRI and reinforcing its role as a complementary, cost-effective modality [58]. In one study, the combination of EAUS and MRI achieved 100% concordance with surgical findings, compared with 87–91% for either modality alone [23]. Comparative information on the respective roles of endoanal ultrasound (EAUS) and magnetic resonance imaging (MRI) in perianal Crohn’s disease is presented in Table 3.

From a practical perspective, EAUS offers unique organizational advantages: it can be used as a first-line tool for rapid triage, repeated at short intervals to monitor therapeutic response, and used intraoperatively for peri-seton management. MRI, on the other hand, remains indispensable for assessing supralevator disease, deep pelvic sepsis, and fibrosis, and is the only modality with validated scoring indices for clinical trials. Rather than competing, the two techniques are complementary. EAUS ensures bedside accessibility and dynamic follow-up, while MRI provides comprehensive mapping and standardized endpoints. Optimal management, therefore, often requires a tailored approach, and in selected cases, the integration of both modalities ensures the most accurate assessment and guidance for therapy in perianal Crohn’s disease.

## 6. Conclusions

Perianal Crohn’s disease is a complex condition where accurate imaging plays a central role in guiding treatment and improving outcomes. Endoanal ultrasound (EAUS) offers high-resolution visualization of the anal canal and sphincter complex, allowing for the precise detection and classification of fistulas and abscesses. Characteristic features such as tract width, bifurcation, CUFS, and the rosary sign support differentiation from cryptoglandular disease, while serial examinations allow close monitoring of therapeutic response and seton management.

Magnetic resonance imaging (MRI), with its panoramic pelvic view and validated scoring systems, remains essential for complex, recurrent, or supralevator disease. EAUS, by contrast, offers unique advantages as a cost-effective, repeatable bedside tool and as the modality of choice for evaluating sphincter integrity, particularly in patients who have undergone prior anorectal surgery. Used together, the two modalities are complementary: EAUS ensures dynamic point-of-care assessment, while MRI provides global mapping and standardized endpoints.

Emerging technologies—including three-dimensional probes, contrast enhancement, Doppler, shear wave elastography, and AI-assisted tract mapping—may further strengthen the role of EAUS, potentially enabling validated activity scores comparable to those established for MRI. A combined and structured approach to both modalities offers the most reliable strategy for tailoring therapy and optimizing long-term outcomes in patients with perianal Crohn’s disease.

Ultimately, the field will benefit from the creation of a validated EAUS-based activity score. Such a tool should be composite, integrating morphological features (e.g., tract width > 4 mm, bifurcation, hyperechoic debris), functional parameters (qualitative Doppler grading and quantitative SWE values), and dynamic changes observed between serial scans. A prospective “Perianal Endosonographic Activity in Crohn’s Evaluation (PEACE) Index,” benchmarked against clinically meaningful outcomes such as durable fistula closure and relapse after biologic withdrawal, would transform EAUS from a primarily anatomical technique into a standardized functional biomarker. The development of such an index represents the logical next step to fully embed EAUS within the treat-to-target framework and to consolidate its role as an indispensable tool in the longitudinal management of perianal Crohn’s disease.

## Figures and Tables

**Table 1:** Structured EAUS report template.

Section	Items to Report
Patient/Indication/Date	Basic demographics, indication for EAUS, and examination date
Technique	Probe MHz; 360° rotation; 2D/3D mode; Doppler/SWE use; patient tolerance
Internal Openings	N, clock face position; distance from anal verge (cm)
Primary Tract Type	Intersphincteric/Transsphincteric/Suprasphincteric/Extrasphincteric; laterality; level (low/mid/high)
Secondary Extensions	Horseshoe (anterior/posterior); contralateral spread; supralevator extension (if suspected)
Collections	N; size (mm); compartments; proximity to skin/mucosa
Activity Markers	Tract width (mm); internal echoes/debris; Doppler grade (0–2); SWE values (m/s or kPa, if available)
Sphincters	IAS/EAS integrity; presence of scars/defects; risk to continence
Devices/Surgery	Setons (number and path); drains; stomas
Synthesis/Impact	Overall impression with Parks classification; actionable next steps (e.g., drainage, seton placement, MRI, interval for repeat EAUS)

EAUS = Endoanal ultrasound; SWE = Shear wave elastography; N = Number (di aperture interne); IAS = Internal anal sphincter; EAS = External anal sphincter; MRI = Magnetic resonance imaging.

**Table 2:** Summary of the relevant literature about typical EAUS signs of pCD.

First Author (Year)	EAUS Sign(s)	Diagnostic Performance
Blom, J. (2011) [30]	Bifurcation/secondary tracts; width > 3 mm; debris	Suggested CD, but without detailed sensitivity/specificity
Zawadzki, A. (2012) [31]	CUFS ^1^	Specificity 98%; Sensitivity 69%; κ = 0.77
Zbar, A.P. (2013) [32]	CUFS ^1^	Specificity 97%; Sensitivity 43%; κ = 0.85
Luglio, G. (2018) [33]	CUFS ^1^ + tract width > 4 mm	Specificity 88–100%; Sensitivity up to 100%; κ = 0.84
de la Portilla, F. (2022) [29]	Rosary sign ^2^	Specificity 71%; Sensitivity 49%; κ = 0.27

^1^ CUFS: hypoechogenic fistula tract with regular hypoechogenic border surrounded by a hyperechogenic area. ^2^ Rosary sign: discontinuous ‘rosary bead’ shaped hypoechoic halo in the intersphincteric space and circumferentially located in the upper half of the anal canal.

**Table 3:** Comparative roles of endoanal ultrasound (EAUS) and magnetic resonance imaging (MRI) in perianal Crohn’s disease.

Feature	EAUS	MRI
Availability and logistics	Outpatient, cost-effective, quick (15–20 min)	Higher cost, limited access, requires scheduling
Contraindications	Minimally invasive, but limited in stenosis or severe anal pain	Non-invasive, but contraindicated in claustrophobia, metal implants, or gadolinium restriction
Field of view	High-resolution imaging of anal canal, sphincter complex, and intersphincteric space	Panoramic multiplanar imaging of entire pelvis, including supralevator and extrapelvic disease
Internal opening detection	Excellent, especially with 3D probes and hydrogen peroxide enhancement	Reliable, but may be less precise than EAUS for fine sphincteric detail
Secondary extensions	Good for low and intersphincteric tracts; limited for very lateral or supralevator disease	Superior for complex, high, and recurrent tracts; comprehensive mapping
Abscess detection	Accurate for perianal and intersphincteric abscesses; limited in deep pelvis	Excellent sensitivity for deep pelvic and supralevator collections
Validated scoring systems	None validated; candidate parameters under study (tract width, echoes, Doppler, SWE)	Several validated indices (Van Assche, MAGNIFI-CD, mVAI) used in trials and monitoring
Role in clinical pathway	First-line, bedside triage, perioperative seton guidance, serial monitoring	Reference standard for complex/recurrent disease, surgical planning, and research endpoints

## Data Availability

Not applicable.

## Abbreviations

The following abbreviations are used in this manuscript:

TNF	Tumor Necrosis Factor
EAUS	Endoanal ultrasound
T2T	Treat-to-target
TPUS	Transperineal ultrasound
MRI	Magnetic resonance imaging
pCD	Perianal Crohn’s disease
CUFS	Crohn’s Ultrasound Fistula Sign
SWE	Shear wave elastography
